# Effect of different concentrations of Trolox^®^ in association with docosahexaenoic acid on equine semen freezing

**DOI:** 10.1590/1984-3143-AR2022-0010

**Published:** 2022-11-28

**Authors:** Cristiane Silva Aguiar, Celso Henrique Souza Costa Barros, William Morais Machado, Ivan Bezerra Allaman, Antônio de Oliveira Leite, Larissa Pires Barbosa, Paola Pereira das Neves Snoeck

**Affiliations:** 1 Universidade Estadual de Santa Cruz, Ilhéus, BA, Brasil; 2 Universidade Federal do Recôncavo da Bahia, Cruz das Almas, BA, Brasil; 3 Centro Universitário INTA, Sobral, CE, Brasil; 4 Haras Pamplona, Cabaceiras do Paraguaçu, BA, Brasil

**Keywords:** antioxidant, fatty acids, lipid peroxidation, cryopreservation, stallion

## Abstract

The aim of this study was to evaluate the association of different concentrations of Trolox^®^ and the addition of a fixed concentration of DHA in the freezing of semen of Mangalarga Marchador stallions. To that end, 16 ejaculates were frozen in the following extenders: E1) BotuCrio^®^ (BC; Control); E2) BC + 50 ngml^-1^ DHA + 30 µM Trolox^®^ (BCDHA30T); E3) BC + 50 ngml^-1^ DHA + 40 µM Trolox^®^ (BCDHA40T); E4) BC + 50 ngml^-1^ DHA + 50 µM Trolox^®^ (BCDHA50T). All the tested extenders were similar in preserving different kinematic parameters, cell functional integrity, compacted DNA, and high and intermediate mitochondrial activity (P>0.05). However, sperm cryopreserved in BCDHA40T showed higher velocities than sperm frozen in the control extender (P<0.05). The 30 µM concentration of Trolox^®^ was worse for sperm motility and the 50 µM concentration of Trolox^®^ did not adequately preserve the structural integrity of the membranes in an extender containing DHA when compared to the BotuCrio^®^ (P<0.05) extender. The use of Trolox^®^ in freezing extenders containing DHA did not maximize the effect of BotuCrio^®^, except for in the case of sperm velocity parameters when at a concentration of 40 µM.

## Introduction

Among Brazil’s national breeds, the Mangalarga Marchador is known for having individuals with sperm of varied cryoresistance (Alvarenga et al., 2005), with a greater number of stallions with low capacity to maintain sperm viability after cryopreservation (Gomes et al., 2002; Murphy et al., 2014). Efforts to improve this semen conservation biotechnique are focused on modifying extenders and protocols, with the aim of increasing sperm longevity and its fertile potential after freezing (Sieme et al., 2016).

Oxidative stress generated in the cryopreservation process and the addition of antioxidants to cooling media (Aguiar et al., 2020), semen freezing (Silva et al., 2008), or to dietary supplements (Domosławska et al., 2018), as a way to protect sperm from damage induced by reactive oxygen species (ROS) has been demonstrated in several studies. Damage induced by ROS production encompasses changes in motility, viability, energy production, and sperm DNA integrity. The antioxidants used act by interrupting the lipid peroxidation chain reaction of sperm membranes in several animal species (Maia and Bicudo, 2009).

Alpha tocopherol is able to stop the lipid peroxidation chain reaction in biomembranes, protecting the cell from damage to the plasma and acrosomal membranes (Sikka, 2004; Nichi, 2009; Towhidi et al., 2013). Trolox^®^ is a water-soluble alpha tocopherol analogue, which, due to this characteristic, has a potent antioxidant property (Wu et al., 1991).

Insufficient amounts of antioxidants in the extender medium can facilitate lipid peroxidation and damage sperm, which are particularly vulnerable (Hatamoto et al., 2006), since the plasma membrane of sperm cells has varying amounts of polyunsaturated fatty acids (PUFAs) (Waterhouse et al., 2006; Wood et al., 2016; Evans et al., 2020). This is similar to docosahexaenoic acid (DHA), which is capable of providing the necessary fluidity for important cellular events to occur, such as the fusion of membranes during the fertilization process. The oxidation of these fatty acids in the sperm plasma membrane decreases its fluidity, increases its permeability, and consequently decreases its fertilization capacity (Lenzi et al., 2000).

Larger amounts of PUFAs in the plasma membrane have been positively correlated with membrane integrity after thawing (García et al., 2011), as well as with increased antioxidant capacity (Martínez-Soto et al., 2013). According to Evans et al. (2020), however, the amount of PUFAs in the plasma membrane of bovine spermatozoa considered good freezers is no different than that of bad freezers.

The association of a PUFA with an antioxidant is important due to the susceptibility of DHA double bonds to the action of ROS (Towhidi and Parks, 2012). Although Aguiar et al. (2020) did not observe the need for the use of an antioxidant in equine semen cooled with extenders containing DHA. Wood et al. (2016) reported that glycerophospholipids and seminolipids are essential in the plasma membrane of equine sperm and in their cellular functions, emphasizing that low concentrations of DHA result in limited replacement of fatty acids in several glycerophospholipids. The importance of this in the stability of the plasma membrane of cryopreserved equine sperm is yet to be examined.

Considering that the association of Trolox^®^ and DHA can improve the effect of semen extenders during freezing, increasing the parameters of sperm viability and fertility, the aim of this study was to evaluate the association of different concentrations of Trolox^®^ and the addition of a fixed concentration of DHA in the freezing of Mangalarga Marchador stallion semen.

## Methods

### Ethical considerations

The present study was approved by the Comitê de Ética na Experimentação Animal (Ethics Committee on Animal Experimentation) CEUA/UESC of the Universidade Estadual de Santa Cruz (State University of Santa Cruz), Ilhéus, Bahia, Brazil under protocol number 004/16.

### Reagents and solutions

All the reagents used were pure reagents for analysis and purchased from the Sigma-Aldrich^®^ company (St. Louis, MO, USA).

DHA and Trolox^®^ were added to the semen extenders after preparing a stock solution. The DHA stock solution was calculated considering its molecular weight of 328.49 g/mol and diluted in 100 mL of 0.05% ethanol solution (76.1058 x 10^-4^ mol/L). Trolox^®^ stock solution was prepared and diluted in Tris-citric acid solution (Wu et al., 1991), considering its molecular weight of 250.29 g/mol (at 10mM; 0.0125g of Trolox^®^ in 5 mL of Tris buffer).

The experimental groups were: E1) BotuCrio^®^ (BC; control); E2) BC + 50 ngml^-1^ of DHA + 30 µM of Trolox^®^ (BCDHA30T); E3) BC + 50 ngml^-1^ of DHA + 40 µM of Trolox^®^ (BCDHA40T); E4) BC + 50 ngml^-1^ of DHA + 50 µM of Trolox^®^ (BCDHA50T).

### Location, animals and collection

The experiment was carried out at stud farms located in the municipality of Cabaceiras do Paraguaçu, Bahia, Northeast, Brazil (latitude 12°32′9″S, longitude 39°11′27″W, altitude 210m). The climate is tropical, with average annual rainfall of 932 mm and average annual temperature of 23.2 ºC. Semen analyses were performed at the Laboratório de Reprodução Animal (Animal Reproduction Laboratory) of UESC.

Four stallions of the Mangalarga Marchador breed, aged between 5 and 7 years old, with a body condition score of 4 were used in the study. Before the beginning of the experiment, all the stallions were subjected to the depletion of their extragonadal sperm reserves, through serial semen collections, for seven days, using a Botucatu artificial vagina (Botupharma, Botucatu, SP, Brazil) and the help of a female in estrus as a mannequin. The ejaculate of each stallion was collected four times, with an interval of 48 hours between collections, obtaining a total of 16 ejaculates.

### Semen processing

After collection, the semen was filtered in a nylon filter (Minitub^®^, Germany) to remove the gel fraction and the ejaculates were evaluated subjectively, macro and microscopically, according to the standards of the Brazilian College of Animal Reproduction (CBRA, 2013) before freezing. Sperm concentration was measured using a Neubauer chamber, after dilution of 1:20 in a sodium citrate solution with 4% formaldehyde. Only ejaculates with a minimum motility of 60%, sperm vigor of 3, and 70% morphologically normal sperm, were frozen.

After collection and evaluation, the semen was diluted 1:2 in a skimmed milk-based medium (BotuSêmen^®^, Botupharma, Botucatu, SP, Brazil) and centrifuged at 600xg for 10 min. The supernatant was discarded, and the pellet was diluted as per the abovementioned experimental groups.

The diluted semen was bottled in 0.25 mL straws, at a concentration of 100x10^6^ motile sperm per mL. The straws were sealed with polyvinyl alcohol and frozen in an automated system (TK4000^®^, TK Technology in freezing, Uberaba, Brazil). The cooling curve used was -0.5 °C per minute from 20.5 °C to 5 °C, then the samples remained in equilibrium for 20 minutes at a temperature of 5 °C and were frozen at a rate of -25 °C/min down to -140 °C to then be immersed in liquid nitrogen (-196 °C) and stored in a cryogenic cylinder.

A straw from each treatment was thawed and its total content was transferred to polyethylene microtubes, which were already heated in a dry bath at 37 ºC. The samples were kept under these conditions and evaluated after 5, 60, and 120 minutes for sperm movement parameters, as follows, to assess sperm longevity after cryopreservation.

### Semen analysis after thawing

Sperm movement of the thawed samples was evaluated by a computerized Sperm Class Analyzer^®^ system (Microptics S.L, v.5.2, Barcelona, Spain). The standards used to adjust the equipment were: 25 images/second at 25 Hz; particle size captured between 4 and 75 µm/m2; the spermatozoa were considered immobile <10 μm/s, slow <45 μm/s, medium from 45 to 90 μm/s, and fast above 90 μm/s. The following parameters were evaluated: Total Motility (TM), Progressive Motility (PM), Linearity (LIN), Straightness (STR), Hyperactives expressed as a percentage (%); Curvilinear Velocity (VCL), Linear Progressive Velocity (VSL), and Average Path Velocity (VAP), expressed in micrometers per second (µm/s); Amplitude of Lateral Sperm Head Displacement (ALH), expressed in micrometers (µm); and Cross Flagellar Beat Frequency (BCF), expressed in Hertz (Hz).

The structural integrity of the plasma and acrosomal membranes was evaluated using a fluorescent microscope (400X; Olympus® CX 31) after staining the sperm with fluorescent dyes of carboxyfluorescein diacetate (CFDA) and propidium iodide (PI), according to the Harrison and Vickers (1990) method. CFDA staining was assessed using the standard set of fluorescein filters, while IP staining was assessed using the standard set of rhodamine filters. Two-hundred sperm were analyzed per sample. The sperm were classified into three subpopulations: structurally intact, with intact plasma and acrosomal membranes (IP-, CFDA+); partially intact, with damaged plasma membrane and intact acrosomal membrane (IP+, CFDA+); total loss of integrity, with damaged plasma and acrossomal membranes (IP+, CFDA-). For the purpose of evaluating the efficiency of the extenders, only the percentage of sperm with intact plasma and acrosomal membranes was considered.

The functional integrity of the plasma membrane was evaluated using the hyposmotic test (HOST) with 50 μL of the sample diluted in 500 μL of a sucrose solution at 100 mOsmol/L. After dilution, samples were first incubated in a dry bath at 37 °C for 30 minutes and then fixed with 250 μL of sodium citrate solution with 4% formaldehyde, and 200 cells were evaluated using a phase contrast microscope (1000x; Olympus^®^ CX 31).

The percentage of cells reactive to the HOST was calculated as follows: HOST% = % changes in the tail region after HOST - % changes in the tail region before HOST, according to the method of Melo and Henry (1999). The tail changes before the test were analyzed using the wet preparation technique, diluting a semen sample in a sodium citrate solution with 4% formaldehyde for subsequent evaluation of sperm morphological changes using a phase contrast microscope (1000x; Olympus^®^ CX 31). Two-hundred sperm were evaluated.

The integrity of sperm chromatin was assessed using the toluidine blue-induced metachromasia technique (Naves et al., 2004). Smears were made with an aliquot of 10 μL of the sample, dried at room temperature and fixed for 1 min in Carnoy's solution (3:1, 75 mL of 100% alcohol + 25 mL of acetic acid) and then in 70% alcohol for 3 min. Hydrolysis proceeded with 4N hydrochloric acid for 15 minutes, washing in distilled water, and drying at room temperature. For staining the smear, 20 μL of 0.025% toluidine blue solution (0.00125 g of toluidine blue in 5 mL of McIlveine solution, pH 4.0) was deposited between the slide and cover slip and 500 cells were evaluated using a phase-contrast microscope (1000x; Olympus^®^ CX 31). Sperms were classified as follows: with compact chromatin (head region stained in light blue); and with decompacted chromatin (head region stained in dark blue or violet).

Mitochondrial activity was evaluated through staining with 3,3'-diaminobenzidine (DAB) according to the technique of Hrudka (1987), whereby 20 μL of the sample was incubated with 20 μL of DAB (1mg/mL PBS) at 37 ºC for 60 minutes, in the absence of light. After incubation, smears were made, fixed in 10% formalin for 10 minutes, washed in distilled water, and air-dried protected from light. Two-hundred cells were evaluated under a phase contrast microscope (1000x; Olympus^®^ CX 31). Cells were classified according to the level of dye deposition on the intermediate piece (IP). In class I, the sperm had fully stained IP (high mitochondrial activity); in class II, sperm had more than 50% of the IP stained (intermediate mitochondrial activity); in class III, they presented less than 50% of the IP stained (low mitochondrial activity); and in class IV, they did not present staining (nonexistent mitochondrial activity).

The level of lipid peroxidation was measured using the thiobarbituric acid (TBA) assay according to the method described by Buege and Aust (1978). Thiobarbituric acid reactive substances (TBARS) were measured in the semen immediately after thawing (spontaneous lipid peroxidation) or after incubation with 0.24 mM FeSO4 at 37°C in a water bath for 15 minutes (iron catalyzed lipid peroxidation or induced lipid peroxidation). In the analysis of spontaneous lipid peroxidation, the content of a 0.25 mL semen straw was added with 0.25 mL of Tris-citric acid buffer (Tris-hydroxymethyl-aminomethane 1.8184 g + citric acid monohydrate 0. 9901 g + distilled water up to 50 mL, with pH 7.4) to obtain a final volume of 0.5 mL. Then, 1 mL of TBA reagent (15% wt:volume trichloroacetic acid, 0.25 N hydrochloric acid, 0.375% wt:volume thiobarbituric acid in distilled water qsp to 100 mL) was added, then 1% (15μL, v:v) of the 50 mM BHT (butylated hydroxytoluene) solution was added. The mixture was boiled for 15 minutes and then cooled in a bath of crushed ice. After cooling, the suspension was centrifuged at 1200 g for 15 minutes. The supernatant was then separated, kept in an ice bath, and the absorbance was measured at 546 nm at 25 °C.

The positive control of this reaction is the analysis of iron-induced or catalyzed lipid peroxidation, which aims to measure the potential of a sample to generate ROS, as follows: in a second sample (250 μL of semen + 250 μL Tris buffer), 50 µL of ferrous sulfate heptahydrate (0.013 g of FeSO4 + 20 mL of 2.4 mM distilled water) and 10 µL of 50 mM ascorbic acid solution (0.08806 g of L-ascorbic acid + 10 mL of distilled water) were added. The sample was incubated in a water bath at 37 °C for 15 minutes, followed by the evaluation of spontaneous lipid peroxidation as described.

Lipid peroxidation analysis was performed using a spectrophotometer (Bio-2000 IL, Bioplus Ltd., São Paulo, BR). The concentration of TBARS was determined by comparing the absorbance of the sample at 546 nm to a standard curve created from malondialdehyde equivalents (MDA) generated by the acid-catalyzed hydrolysis of 1,1,3,3-tetramethoxopropane. The results were expressed in nmol of TBARS/mL.

## Statistical analyses

The experimental design was in randomized blocks, considering each stallion as a block. Analysis of variance was used to test differences between the treatments. The Tukey test was used to compare means, with a significance level of 5%. All assumptions were tested and when violated the boxcox transformation was used (y^ʹ^ = (y^λ-^1)/λ) through the “boxcox” function of the MASS package version 7.3-41 (Venables and Ripley, 2002). The transformed variables were: DAB I, III, and VI. All analyses were performed using R Core Team (The R Project for Statistical Computing, 2016). The data were presented in the tables as means, even those that underwent transformation for statistical analysis.

## Results

The extender containing DHA and 30 µM Trolox^®^ was inferior to BotuCrio^®^ in preserving total motility (P<0.05). VCL, VSL, and VAP were higher in the extender containing DHA and 40 µM Trolox^®^ than in BotuCrio^®^ (P<0.05). Sperm cryopreserved in extenders containing DHA and different concentrations of Trolox^®^ had higher ALH than sperm cryopreserved in the control extender (P<0.05). The four extenders tested similarly preserved (P>0.05) the functional integrity of the plasma membrane, the integrity of the chromatin, and the high and intermediate mitochondrial activity. Sperm frozen in the extender containing DHA and 30 or 40 µM of Trolox^®^ had a lower percentage of cells with low mitochondrial activity compared to BotuCrio^®^ (P<0.05). The extender with DHA and 50 µM of Trolox^®^ was inferior in preserving the structural integrity of sperm membranes when compared to BotuCrio^®^ (P<0.05, Table 1).

**Table 1:** Sperm viability parameters after freezing equine semen in extender added with DHA and different concentrations of Trolox^®^.

**PARAMETERS**	**EXTENDER**				**SEM**	**P-value**
	**BC**	**BC DHA 30T**	**BC DHA 40T**	**BC DHA 50T**		
**TM(%)**	40.60^a^	28.40^b^	34.30^ab^	35.00^ab^	2.85	0.04
**PM(%)**	8.10	6.90	8.80	8.70	1.14	0.61
**VCL (µm/s)**	31.60^b^	35.20^ab^	37.70^a^	35.80^ab^	1.27	0.01
**VSL (µm/s)**	12.70^b^	14.30^ab^	16.30^a^	14.50^ab^	0.94	0.08
**VAP (µm/s)**	17.20^b^	19.20^ab^	21.20^a^	19.40^ab^	0.95	0.04
**LIN (%)**	39.10	40.40	41.00	39.80	1.28	0.75
**STR (%)**	73.70	73.80	75.50	74.80	1.23	0.71
**ALH (µm)**	2.50^b^	2.70^a^	2.70^a^	2.70^a^	0.05	0.00
**BCF (Hz)**	13.00	12.50	12.70	12.40	0.29	0.48
**IP-CFDA+ (%)**	38.80^a^	32.90^ab^	33.40^ab^	31.80^b^	1.86	0.05
**HOST(%)**	35.40	24.10	28.60	25.10	3.26	0.07
**DNAc (%)**	84.30	85.00	85.20	84.30	1.60	0.97
**DNAd (%)**	15.70	15.00	14.80	15.70	1.60	0.97
DABI (%)*	76.30	82.00	79.00	78.90	5.73*	0.46
**DABII (%)**	8.10	6.00	3.90	5.00	1.21*	0.10
DABIII (%)^*^	5.20^a^	2.30^b^	2.30^b^	2.50^ab^	0.65*	0.00
DABIV (%)^*^	10.50	9.40	10.7	9.10	3.76*	0.12
**TBARSe (nMol/mL)**	0.14	0.07	0.04	0.06	0.05	0.43
**TBARSc (nMol/mL)**	0.52^a^	0.03^b^	0.09^b^	0.00^b^	0.02	0.00

E1) BotuCrio® (BC; control); E2) BC + 50 ngmL-1 DHA (BCDHA) + 30 µM Trolox® (BCDHA30T); E3) BCDHA + 40 µM Trolox® (BCDHA40T); E4) BCDHA + 50 µM Trolox® (BCDHA50T). Total motility (TM, %), progressive motility (PM, %), %), curvilinear velocity (VCL, µm/s), straight linear velocity (VSL, µm/s), average path velocity (VAP, µm/s), linearity (LIN, %), rectilinearity (STR, %), head displacement (ALH, µm) and tail beat frequency (BCF, Hz). Structural (IP-CFDA+) and functional integrity (hypoosmotic test, HOST) post-thaw. Compacted DNA (DNAc); Decompacted DNA (DNAd). High mitochondrial activity (DAB I); Intermediate mitochondrial activity (DAB II); Low mitochondrial activity (DABIII); No mitochondrial activity (DAB IV). Concentration of thiobarbituric acid reactive substances (TBARS) in spontaneous lipoperoxidation (TBARSe) and FeSO4 catalyzed lipoperoxidation (TBARSc). ^ab^Superscript letters indicate differences within the line (P<0.05). *Inferences for such variables were made after transformation.

All the tested extenders controlled spontaneous lipid peroxidation similarly (P>0.05). However, for induced lipid peroxidation, treatments with DHA and Trolox^®^ at all the concentrations reduced the production of TBARS compared to BotuCrio^®^ (P<0.05, Table 1).

The tested extenders similarly preserved several sperm movement parameters (motility, VCL, VSL, and VAP) during the two-hour incubation period for the sperm longevity assessment (P>0.05). However, it was noticed that after 60 minutes of incubation, the ALH of sperm cryopreserved in BCDHA50T was lower than in samples cryopreserved in BotuCrio^®^ (P<0.05; Table 2).

**Table 2:** Sperm kinematic parameters after 60 and 120 minutes of incubation at 37º C after freezing in extender containing DHA and Trolox^®^.

**Kinematics Parameters 60 min**	**EXTENDER**				**SEM**	**P-value**
	**BC**	**BC DHA 30T**	**BC DHA 40T**	**BC DHA 50T**		
TM(%)	20.20	23.00	22.40	22.10	2.30	0.84
VCL (µm/s)	30.90	32.40	33.00	31.00	1.32	0.63
VSL (µm/s)	12.80	13.20	13.70	13.50	0.79	0.85
VAP (µm/s)	17.10	17.50	18.10	17.30	0.80	0.84
ALH (µm)	2.60^a^	2.60^ab^	2.50^ab^	2.40^b^	0.05	0.04
**Kinematics Parameters 120 min**	**BC**	**BC DHA 30T**	**BC DHA 40T**	**BC DHA 50T**		
TM(%)	11.20	11.40	13.70	11.40	1.07	0.58
VCL (µm/s)	28.50	28.40	29.40	24.90	1.26	0.07
VSL (µm/s)	11.80	11.50	12.10	10.20	0.70	0.24
VAP (µm/s)	15.80	15.40	16.30	14.00	0.72	0.16
ALH (µm)	2.10	2.10	2.20	2.00	0.13	0.79

E1) BotuCrio^®^ (BC; control); E2) BC + 50 ngmL-1 DHA (BCDHA) + 30 µM Trolox^®^ (BCDHA30T); E3) BCDHA + 40 µM Trolox^®^ (BCDHA40T); E4) BCDHA + 50 µM Trolox^®^ (BCDHA50T). Total motility (TM, %), curvilinear velocity (VCL, µm/s), straight linear velocity (VSL, µm/s), average path velocity (VAP, µm/s), head displacement (ALH, µm). ^ab^Superscript letters indicate differences within the line (P<0.05).

## Discussion

The 30 µM concentration of Trolox^®^ impaired total sperm motility and the 50 µM concentration of Trolox^®^ did not adequately preserve the structural integrity of the membranes in extenders containing DHA compared to BotuCrio^®^. The use of Trolox^®^ in freezing extenders containing DHA did not maximize the effect of the control extender, although sperm velocity parameters were positively impacted with the addition of 40 µM.

There are controversial reports in the literature regarding the benefits of adding alpha tocopherol to semen extenders. In some studies, this antioxidant improved stallion (Hrudka, 1987; Silva et al., 2008), ram (Sarlós et al., 2002), and boar (Breininger et al., 2005) sperm motility, or had no effect on the motility of ram (Upreti et al., 1997) or stallion sperm (Ball et al., 2001; Aguiar et al., 2020). Other authors have already indicated an inverse relationship between the rate of lipid peroxidation and sperm motility, in addition to finding greater amounts of antioxidants in sperm with better quality movement (Kasimanickam et al., 2006; Kao et al., 2008). It is known that lipid peroxidation products affect mitochondrial integrity and energy supply for movement (Sabeti et al., 2016), which may have positively influenced the speed of samples frozen in an extender containing 40µM of Trolox^®^ and the low percentage of sperm with low mitochondrial activity in all samples cryopreserved in extenders containing DHA and Trolox.

Wassall and Stillwell (2009) reported that there is a change in the ratio of ω-6:ω-3 after the incorporation of DHA in sperm membranes through its addition to extenders (Towhidi and Parks, 2012) and of PUFA:saturated fatty acids (Nasiri et al., 2012), reflecting improved fluidity and, consequently, motility (Connor et al., 1998). It is known that the incorporation of DHA in the plasma membrane is responsible for increasing its flexibility and protecting the sperm from the harmful effects of freezing (Nasiri et al., 2012).

Several important molecules involved in the incorporation of DHA into cell membranes and in the production of glycerophospholipids containing DHA, such as LPAAT3, Mfsd2a, and AdipoR1, have been identified (Nguyen et al., 2014; Rice et al., 2015; Iizuka-Hishikawa et al., 2017; Shindou et al., 2017). However, ways of regulating DHA incorporation and the exact functions of DHA in membrane glycerophospholipids (Hishikawa et al., 2017) and its importance in the stability of the plasma membrane of cryopreserved equine spermatozoa (Wood et al., 2016) remain unclear.

The beneficial effect of dietary supplementation with DHA on semen quality has been reported in humans (Safarinejad et al., 2010), swine (Mitre et al., 2004), horses (Harris et al., 2005), mice (Roqueta-Rivera et al., 2010), and cattle (Gholami et al., 2010). Kaeoket et al. (2010) found that the inclusion of different concentrations of fish oil as a source of DHA in semen extenders resulted in greater viability, progressive motility, and acrosomal integrity. However, 100 ngmL^-1^ of DHA in bull semen freezing extenders reduced post-thaw semen quality, which did not occur in samples frozen with the lowest concentrations (Towhidi and Parks, 2012). The use of DHA in semen extenders has positive results; however, its association with alpha tocopherol for freezing equine semen will depend on the concentrations used.

The inclusion of 30 and 50 µM of Trolox^®^ in extenders containing a fixed concentration of DHA negatively affected motility and plasma and acrosomal membrane integrity, respectively. The negative effects of Trolox on motility have already been observed by Sicherle et al. (2006) when they used concentrations equal to or above 150 µM in sheep semen freezing extenders. It is known that the beneficial effects of adding antioxidants to semen extenders are obtained depending on the concentration used and the composition of the medium. The hydroxyl radical neutralizing capacity of Trolox^®^ is diminished when it is at high concentrations, possibly related to its interaction with the hydroxyl radical and other oxygen radicals produced in the presence of H_2_O_2_ and Cu^2+^, leading to the formation of many radicals at the same time. Tocopherols can reduce Fe^3+^ to Fe^2+^ and Cu^2+^ to Cu^+^, which exert a pro-oxidant effect (Halliwell and Gutteridge, 2015). Thus, the use of vitamin E as an antioxidant can be favorable or unfavorable, depending strictly on its *in vitro* concentration (Bolle et al., 2002). Considering this aspect, it is not always true that the use of higher amounts of antioxidant substances proportionally increases their antioxidant capacity (Cao and Cutler, 1993). In addition, high concentrations can be deleterious, considering the physiological role of ROS in important cellular physiological processes and concentrations of antioxidant substances below the necessary amount may be insufficient (Nichi, 2003).

All the tested extenders similarly preserved functional integrity and more than 80% of sperm with compact chromatin, despite reports that lowering the temperature during refrigeration and freezing damage these structures (McCarthy and Meyers, 2011). It is also known that oxidative stress is among the main causes of DNA fragmentation and infertility (Agarwal and Said, 2003). Evenson and Jost (2001) reported lower fertile potential when the ejaculate of stallions has more than 30% of sperm with fragmented DNA compared to animals that had around 15%. A DNA fragmentation around 25% can result in a pregnancy rate of 39%, while samples with 12% of sperm with DNA fragmentation can guarantee a pregnancy rate per estrous cycle of 82% (Brinsko et al., 2005). The DNA decompaction observed in the present study was less than 20% in samples cryopreserved in different extenders. The integrity of sperm DNA is of great importance as an expression of fertility potential (Fraser, 2004).

Mitochondria are the main source of ATP to ensure sperm metabolism (Peña et al., 2009). About 60% of the generated ATP is consumed as a driving force, while the remainder is consumed in the phosphorylation and dephosphorylation of substrates (Hammerstedt et al., 1990). All the treatments similarly preserved high and intermediate mitochondrial activity, which is important, as one of the ways of altering motility through the presence of excessive concentrations of ROS may be the alteration in mitochondrial function (Jonathan et al., 2015). Furthermore, there is a strong correlation between high mitochondrial membrane potential and fertile sperm potential (Costa et al., 2015).

There was no difference between the treatments in the assessment of spontaneous lipid peroxidation. However, the addition of Trolox^®^ promoted a reduction in the formation of TBARS in the analysis of lipid peroxidation catalyzed by FeSO^4^. In this assessment, no detectable amounts of TBARS were observed in the extender that contained 50 ngmL^-1^ of DHA and 50 µM of Trolox^®^, this being the highest concentration of Trolox^®^ studied. The other extenders had low TBARS values when compared to the control extender, demonstrating that the proposed Trolox^®^ concentrations were able to control lipid peroxidation as expected.

In the study of iron-catalyzed lipid peroxidation, considered an oxidative stress condition, extenders containing Trolox^®^ were more effective at reducing the amounts of TBARS than the control extender, possibly by inhibiting the chain propagation of lipid peroxidation, thus corroborating the studies of Sicherle et al. (2011). The antioxidant effect of Trolox^®^ is similar to that of Vitamin E, both of which act in the removal of peroxyl radicals, interrupting the lipid peroxidation chain reaction (Albertini and Abuja, 1999; Brigelius-Flohé and Traber, 1999). Both have the same absorption capacity for these radicals due to their same functional structure (Cao et al., 1993), as they neutralize two peroxyl radicals per molecule (Burton et al., 1983). However, comparisons between lipid peroxidation assessments in different studies are difficult to make due to a wide variety of methodologies, sperm concentrations in samples, extenders used, and individual variation between animals and species (Sicherle et al., 2011).

There was no difference for the sperm kinematics variables evaluated in the 120-minute incubation time at 37 ºC, except for ALH at 60 minutes; this parameter showed the highest value in the spermatozoa cryopreserved in BotuCrio^®^ and the lowest in the extender with the highest concentration of Trolox^®^. ALH is correlated with the ability to penetrate the zona pellucida of the oocyte, thus being one of the parameters that influences the fertilization process (Verstegen et al., 2002).

## Conclusion

The addition of 40 µM of Trolox^®^ and 50 ngmL^-1^ of DHA to BotuCrio^®^ can be recommended as it preserved different kinematic parameters, structural and functional integrity, DNA, mitochondrial activity, control of lipid peroxidation, and sperm longevity, in addition to maximizing the speed parameters.
